# A Retrospective Observational Study: Is Absolute Lymphocyte Count a Prognostic Marker in COVID-19?

**DOI:** 10.7759/cureus.16554

**Published:** 2021-07-22

**Authors:** Mansoor Zafar, Muhammad Shahbaz, Mangala Karkhanis, Mohamed Abdelbagi, Opeyemi A Makanjuola, Bipin Pun, Ratan S Randhawa, Frederic Cuison, Dana Safarova, Oluwamayowa Ojofeitimi, Kamal Lawrence, Mariya Farooq, Reem Eldebri, Saba Alam, Lucinda Barry, Alisha Khanna, Karuna Subba, Amr Elyasaky, Hesam A Nooredinvand, Manivannan Periasamy, Bolurin A Adekunle, Zahra Maryam, Bao Khuu, Johannes Hegner, Andreia Esteves Morete, Mirej Patel, Gjulio Ciroi, Ubaid Ur Rehman, Jabeen Hsiao, Maaryah J Zafar, Nadiyah Zafar, Bianca A Lazau, Najam-us-Saher Rizvi, Steve Moran, William A O'Neill, Viktoriya Clarke, Stefano Berliti, Athanasios Nakos, Tila Muhammad, Osei Kankam, Mark Whitehead, Ellie M Giddings, Simon Merritt, Umesh Dashora

**Affiliations:** 1 Gastroenterology and General Internal Medicine, Conquest Hospital, East Sussex Healthcare NHS Trust, St. Leonards-on-Sea, GBR; 2 General Internal Medicine, Conquest Hospital, East Sussex Healthcare NHS Trust, St. Leonards-on-Sea, GBR; 3 Emergency Medicine, Conquest Hospital, East Sussex Healthcare NHS Trust, St. Leonards-on-Sea, GBR; 4 Cardiology, Conquest Hospital, East Sussex Healthcare NHS Trust, St. Leonards-on-Sea, GBR; 5 Family and Community Medicine, The London and South East Postgraduate Medical and Dental Education, London, GBR; 6 Gastroenterology, Conquest Hospital, East Sussex Healthcare NHS Trust, St. Leonards-on-Sea, GBR; 7 Family Medicine, Conquest Hospital, East Sussex Healthcare NHS Trust, St. Leonards-on-Sea, GBR; 8 Geriatrics, Conquest Hospital, East Sussex Healthcare NHS Trust, St. Leonards-on-Sea, GBR; 9 Gastroenterology and Hepatology, Conquest Hospital, East Sussex Healthcare NHS Trust, St. Leonards-on-Sea, GBR; 10 Medicine, University of Debrecen, Debrecen, HUN; 11 Medicine, Norfolk and Norwich University Hospitals NHS Foundation Trust, Norwich, GBR; 12 Department of Nuclear Medicine, Sunnybrook Health Sciences Centre, Toronto, CAN; 13 Medicine, Universitatea din Oradea, Oradea, ROU; 14 Nursing, Conquest Hospital, East Sussex Healthcare NHS Trust, St. Leonards-on-Sea, GBR; 15 Dermatology, Aga Khan Health Services, Aga Khan Development Network, Karachi, PAK; 16 Microbiology, Eastbourne District General Hospital, Eastbourne, GBR; 17 Acute Medicine, Conquest Hospital, East Sussex Healthcare NHS Trust, St. Leonards-on-Sea, GBR; 18 Respiratory Medicine/Acute Medicine, Conquest Hospital, East Sussex Healthcare NHS Trust, St. Leonards-on-Sea, GBR; 19 Respiratory Medicine, Conquest Hospital, East Sussex Healthcare NHS Trust, St. Leonards-on-Sea, GBR

**Keywords:** stata version 16 (statacorp texas), covid-19, severe acute respiratory syndrome coronavirus-2 (sars-cov-2), lymphocyte count, treat as positive (tap), restricted cubic splines, firth logistic regression models for mortality, lasso model, alcohol-related liver disease, copd: chronic obstructive pulmonary disease

## Abstract

Aim

Our study aimed to find a correlation between low absolute lymphocyte count and COVID-19-related mortality.

Methods

This study followed a retrospective observational cohort design to analyze the data of patients who presented with symptoms and signs of severe acute respiratory syndrome coronavirus 2 (SARS-CoV-2), at the Conquest Hospital and Eastbourne District General Hospital in East Sussex, United Kingdom, between February 10, 2020 and May 1, 2020, retrospectively. Survival and mortality for the first 30 days and comorbidities were analyzed for all patients who were tested for COVID-19 irrespective of swab results and had blood lymphocyte levels taken at the time of their visit to the ED and their data were analyzed for statistical significance.

Results

A total of 1226 patients had SARS-CoV-2 RNA identification swabs taken between February 10, 2020 and May 1, 2020. A cohort of 742 patients of these patients tested for COVID-19 also had blood lymphocyte levels measured.

Overall, the lymphocyte count did not differ significantly between patients suspected to have COVID-19 infection with either positive or negative COVID-19 swab results.

The lymphocyte count, however, was significantly lower in those who died from COVID-19 (p < 0.001) but when comorbidities were analyzed, we found an association between an increased number of comorbidities and a significantly decreased lymphocyte count.

Conclusion

Once adjusted for comorbidities, the lymphocyte count had no association with COVID-19 infection and mortality.

## Introduction

The 21st century has witnessed a global pandemic of the COVID-19 phenomenon, that has wreaked havoc worldwide issuing lockdowns, panic, and disaster. It is an illness that mainly affects the respiratory system and is caused by the severe acute respiratory syndrome coronavirus 2 (SARS-CoV-2) virus. Information about the prognostic markers is limited. Lymphopenia is defined as a lymphocyte count level less than 1.5 x10^9/l, and several studies suggest an association of COVID-19 with lymphopenia [1,2]. Other studies have focused on lymphocyte count mainly in the ICU during acute admission [3]. However, there is minimal information available about the level of lymphopenia or lymphocyte count that is strongly associated with increased mortality due to COVID-19. The role of comorbidity and other confounding variables is not clear [4].

The majority of the first studies are based on the initial days of the pandemic originating in Wuhan, China [1,2,5]. Soon with the global pandemic, there were studies done highlighting the global implications of the pandemic [3,6,7]. An additional global challenge for clinicians in an acute care setting is the reported studies suggesting 70% sensitivity of the COVID-19 swab test. Studies have reported a pre-test probability of 50% and the post-test probability with a negative test found to be 23%. This suggests it would be far too high to assume someone is not infected [6].

We analyzed a cohort of patients admitted to ED with query COVID-19 infection who had COVID-19 swabs done and had blood lymphocyte count requested; irrespective of a stay in medical wards or the ICU, and analyzed the overall mortality over 30 days, as well its association with comorbidities. To our knowledge, this appears to be the first attempt to analyze the significance of associated comorbidities with various lymphocyte levels and mortality.

Aim of the study

With succeeding waves of the pandemic, we aimed to further characterize the level of lymphocyte count, which is an inexpensive, yet quickly available biomarker, and the risk of mortality with COVID-19, to add to the work done in China [1,2], and the United States of America [3].

## Materials and methods

Methodology

This was a retrospective study approved by our local audit department (East Sussex Healthcare NHS Trust). Data were collected from the electronic medical record system and analyzed. COVID-19 swab results were obtained between February 10, 2020 and May 1, 2020, from the Department of Microbiology Laboratory, for patients being tested at the Conquest Hospital and Eastbourne District General Hospital in East Sussex, United Kingdom. All the relevant blood tests, comorbidities, hospital admissions, and other relevant information were collected from hospital records. A total of 1226 patients had SARS-CoV-2 RNA identification swabs taken between February 10, 2020 and May 1, 2020. A cohort of 742 patients also had a blood test for lymphocyte count through a full blood count (FBC) also known as complete blood count (CBC).

Patients with negative swab results who had features consistent with a diagnosis of COVID-19 including fever, diarrhea, cough, and chest X-ray infiltrates were classified as if COVID-19 positive and were defined as "Treat as Positive" (TAP). This helped to eliminate the discrepancy associated with false-positive or false-negative swab results [6]. 

All the patients who had positive COVID-19 swab results or who were TAP were managed as per NHS England guidelines (publication approval reference: 001559) [7]. This study included only those patients who had both swabs and blood lymphocyte levels tested, including those who were admitted and treated or discharged from the ED. Patients who had swabs taken but did not have blood lymphocyte levels taken were excluded. Comorbidities of patients in the above-mentioned groups were collected from electronic records and analyzed.

Statistical Analyses

The categorical variables were expressed in terms of frequency and percentages and were compared using the chi-square test or Fisher’s exact test. Continuous variables were described as mean (SD) or median (interquartile range [IQR]) and were compared between groups using a two-sample t-test. Spearman correlation was used for correlation assessment. Mortality was assessed using logistic regression models. Lymphocyte count was log-transformed and two severe outliers were removed to give a normal distribution before inclusion in the models. Results are presented as the OR associated with a 20% increase in lymphocyte count. An adjustment was made for patient characteristics and comorbidities by including them as covariates. As the prevalence for some covariates was low, we used a penalized model (Firth logistic regression) to deal with any possible bias due to sparse data [8]. We tested for a difference in effect with age by fitting an interaction term using age as a continuous variable. A probability of obtaining results p-value < 0.05 was taken to be significant. Non-linearity was assessed using restricted cubic splines. All statistical analysis was done using Stata version 16 (StataCorp, Texas).

## Results

A total of 742 patients were tested for SARS-CoV-2 RNA with a swab test and had blood tests for lymphocyte count. The median age was 72 years and the age range was 1-101 years.

Of those suspected to have COVID-19 clinically 112 patients (15.1%) were swab positive and 630 (84.9%) patients were swab negative. Patients with swab-positive results were significantly more likely to be ever smokers and to have comorbidities. Lymphocyte count did not differ significantly between swab-negative and swab-positive patients (Table 1).

**Table 1:** Patient characteristics and comorbidities. ALD: Alcohol liver disease; CLD: Chronic liver disease; COPD: Chronic obstructive pulmonary disease; CXR: Chest X-ray; DMx1: Diabetes mellitus type 1; DMx2: Diabetes mellitus type 2; Frailty: Dalhousie Frailty (Rockwood) score of 4 or more classified as frail; IHD: Ischemic heart disease; ILD: Interstitial lung disease; IQR: Interquartile range; N: Number of cases; PE: Pulmonary embolism.

Variable	Swab negative, N = 630	Swab positive, N = 112	Total	P-value
Age, years	68.4 (19.7)	70.9 (17.6)	68.8 (19.4)	0.21
Sex, % male (N)	47.9% (302)	56.3% (63)	49.2% (365)	0.11
Ever smoker, % (N)	3.9% (24)	16.5% (18)	5.8% (42)	<0.001
CXR infiltrates, % (N)	34.1% (215)	56.3% (63)	37.5% (278)	<0.001
Comorbidities				
Diabetes, % (N) Type 1/Type 2	0.5% (3) 2.6% (16)	0.9% (1) 5.5% (6)	0.6% (4) 3.1% (22)	0.48 0.13
Diarrhea, % (N)	9.1% (57)	29.5% (33)	12.1% (90)	<0.001
IHD, % (N)	17.2% (105)	29.4% (32)	19.1% (137)	0.003
Asthma/COPD/ILD % (N)	10.3% (63)	21.1% (23)	12.0% (86)	0.001
Hypertension, % (N)	10.3% (63)	19.3% (21)	11.7% (84)	0.007
Dementia, % (N)	8.4% (51)	20.2% (22)	10.2% (73)	<0.001
Frailty, % (N)	12.0% (73)	22.9% (25)	13.6% (98)	0.002
ALD-CLD, % (N)	1.5% (9)	1.8% (2)	1.5% (11)	0.68
Malignancy, % (N)	2.6% (16)	14.7% (16)	4.5% (32)	<0.001
PE, % (N)	1.0% (6)	3.7% (4)	1.4% (10)	0.051
Lymphocyte count, 10^9^/ L*	1.12 [0.71-1.68]	1.04 [0.63-1.5]	1.12 [0.71-1.66]	0.27
Lymphocyte count < 1.5x10^9^/ L	69.8% (440)	75.0% (84)	70.6% (524)	0.27

Of the 742 patients, 584 (78.7%) were alive after 30 days, while 158 (21.3%) had died. Patients who died were significantly older (79 vs. 66 years) and more likely to be male (58% vs. 47%), smokers (26% vs. 0.4%), and to have co-morbidities (Table 1). Mortality rates were 33.9% (38/112) in swab-positive patients and 19.1% (120/630) in swab-negative patients. Swab-positive patients who died were more likely to be smokers and had higher rates of comorbidity (Table 1). Lymphocyte counts were significantly lower in patients who died (1.22 vs. 0.76) in the total population and non-significantly lower in the swab-positive population (Table 2).

**Table 2:** Patient characteristics and comorbidities by mortality. ALD: Alcohol liver disease; CLD: Chronic liver disease; COPD: Chronic obstructive pulmonary disease; CXR: Chest X-ray; DMx1: Diabetes mellitus type 1; DMx2: Diabetes mellitus type 2; Frailty: Dalhousie Frailty (Rockwood) score of 4 or more classified as frail; IHD: Ischemic heart disease; ILD: Interstitial lung disease; IQR: Interquartile range; N: Number of cases; PE: Pulmonary embolism.

		All patients			Positive patients		
Variable		Alive	Deceased	P-value	Alive	Deceased	P-value
N		N = 584	N = 158		N = 74	N = 38	
Age	Years	66.1 (20.0)	78.9 (12.1)	<0.001	66.4 (18.6)	79.7 (11.1)	<0.001
Male Sex	% (N)	46.8% (273)	58.2% (92)	0.01	52.7% (39)	63.2% (24)	0.29
Ever smoker	% (N)	0.4% (2)	25.6% (40)	<0.001	0% (0)	48.7% (18)	<0.001
CXR infiltrates	% (N)	30.5% (178)	63.3% (100)	<0.001	52.7% (39)	63.2% (24)	0.29
Comorbidities							
DMx1	% (N)	0.4% (2)	1.3% (2)	0.21	1.4% (1)	0 (0)	1.00
DMx2	% (N)	0.2% (1)	13.6% (21)	<0.001	0% (0)	16.2% (6)	0.001
Diarrhea	% (N)	12.2% (71)	12.0% (19)	0.96	25.7% (19)	36.8% (14)	0.22
IHD	% (N)	6.2% (35)	65.4% (102)	<0.001	4.2% (3)	78.4% (29)	<0.001
Asthma/COPD/ILD	% (N)	2.8% (16)	44.9% (70)	<0.001	1.4% (1)	59.5% (22)	<0.001
Hypertension	% (N)	4.6% (26)	37.2% (58)	<0.001	2.8% (2)	51.4% (19)	<0.001
Dementia	% (N)	4.1% (23)	32.1% (50)	<0.001	5.6% (4)	48.7% (18)	<0.001
Frailty	% (N)	4.3% (24)	47.4% (74)	<0.001	2.8% (2)	62.2% (23)	<0.001
ALD-CLD	% (N)	0 (0%)	7.1% (11)	<0.001	0% (0)	5.4% (2)	0.11
Malignancy	% (N)	0 (0%)	20.5% (32)	<0.001	0% (0)	43.2% (16)	<0.001
PE	% (N)	0 (0%)	6.5% (10)	<0.001	0% (0)	10.8% (4)	0.012
Lymphocyte count*	10^9^/L	1.22 [0.76-1.76]	0.76 [0.52-1.09]	<0.001	1.17 [0.72-1.63]	0.76 [0.58-1.25]	0.057
Lymphocyte count <1.5x10^9^/L	% (N)	66.1% (386)	87.3% (138)	<0.001	71.6% (53)	81.6% (31)	0.36
Covid swab positive	% (N)	12.7% (74)	24.1% (38)	<0.001	-	-	-

We analyzed confounding variables that may have contributed to this finding. Lymphocyte count decreased with age (Spearman's Rho = -0.38; p < 0.001) and was lower in men than women (Table 3). Lymphocyte count was also significantly decreased in those with all comorbidities except in those patients with diarrhea or type 1 diabetes (Table 3). After adjustment for comorbidities, however, there was no significant association of lymphocyte count with mortality in the total population or in swab-positive patients (Table 4).

**Table 3:** Median lymphocyte count [IQR] by sex and comorbidities. ALD: Alcohol liver disease; CLD: Chronic liver disease; COPD: Chronic obstructive pulmonary disease; CXR: Chest X-ray; DMx1: Diabetes mellitus type 1; DMx2: Diabetes mellitus type 2; Frailty: Dalhousie Frailty (Rockwood) score of 4 or more classified as frail; IHD: Ischemic heart disease; ILD: Interstitial lung disease; IQR: Interquartile range; N: Number of cases; PE: Pulmonary embolism.

	No	Yes	P-value
Male sex	1.15 (0.62-1.78) 377	1.06 (0.62-1.54) N = 365	0.03
Diarrhea	1.12 [0.70-1.67] N = 652	1.12 [0.73-1.65] N = 90	0.63
IHD	1.19 [0.73-1.75] N = 582	0.76 [0.47-1.09] N =1 37	<0.001
Asthma/COPD/ILD	1.18 [0.72-1.75] N = 633	0.71 [0.49-0.90] N = 86	<0.001
Hypertension	1.17 [0.71-1.74] N = 635	0.76 [0.59-1.05] N = 84	<0.001
Dementia	1.16 [0.71-1.70] N = 646	0.76 [0.59-1.06] N = 73	<0.001
Frailty	1.18 [0.71-1.74] N = 621	0.77 [0.59-1.08] N = 98	<0.001
Ever smoker	1.15 [0.71-1.7] N = 677	0.75 [0.60-0.88] N = 42	<0.001
ALD/CLD	1.12 [0.70-1.69] N = 708	0.78 [0.59-0.87] N = 11	0.03
Malignancy	1.13 [0.70-1.69] N = 687	0.76 [0.62-0.91] N = 32	0.003
PE	1.12 [0.7-1.68] N = 708	0.67 [0.58-0.88] N = 10	0.02
DMx2	1.12 [0.71-1.69] N = 696	0.76 [0.43-0.92] N = 22	0.01
DMx1	1.11 [0.69-1.66] N = 714	1.12 [0.78-1.66] N = 4	0.85

**Table 4:** Firth logistic regression models for mortality by lymphocyte count. Model 2: Adjusted for age, sex, ever smoking, and swab positivity. Model 3: Adjusted for age, sex, ever smoking, swab positivity, and comorbidities.

	All patients		Swab-positive patients	
	OR (95% CI)	P value	OR (95% CI)	P value
Unadjusted	0.84 (0.79-0.88)	<0.0001	0.94 (0.85-1.05)	0.31
Model 2	0.89 (0.84-0.95)	<0.0001	1.05 (0.90-1.22)	0.54
Model 3	0.96 (0.88-1.04)	0.29	1.03 (0.81-1.32)	0.79

**Figure 1:**
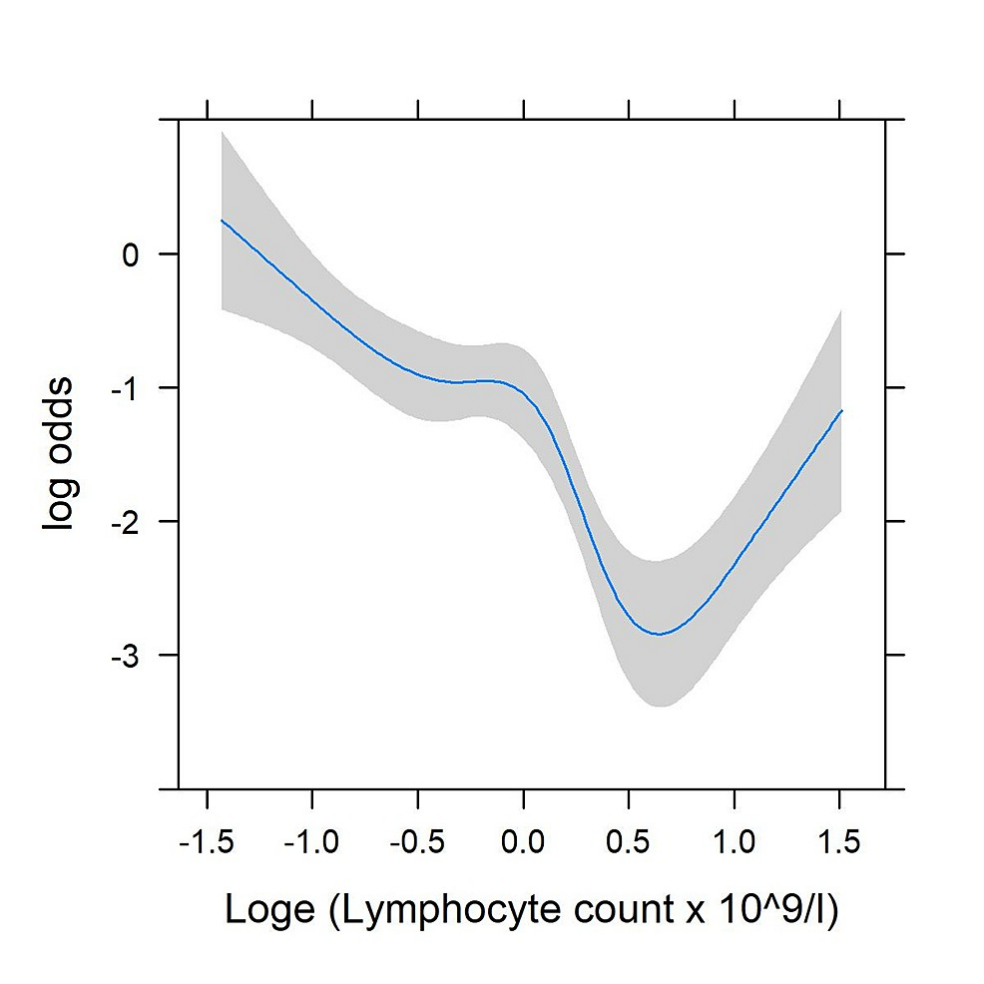
Restricted cubic splines for the non-linear association of lymphocyte count with mortality; unadjusted. Log: Logarithm Loge: Exponent of the log

**Figure 2:**
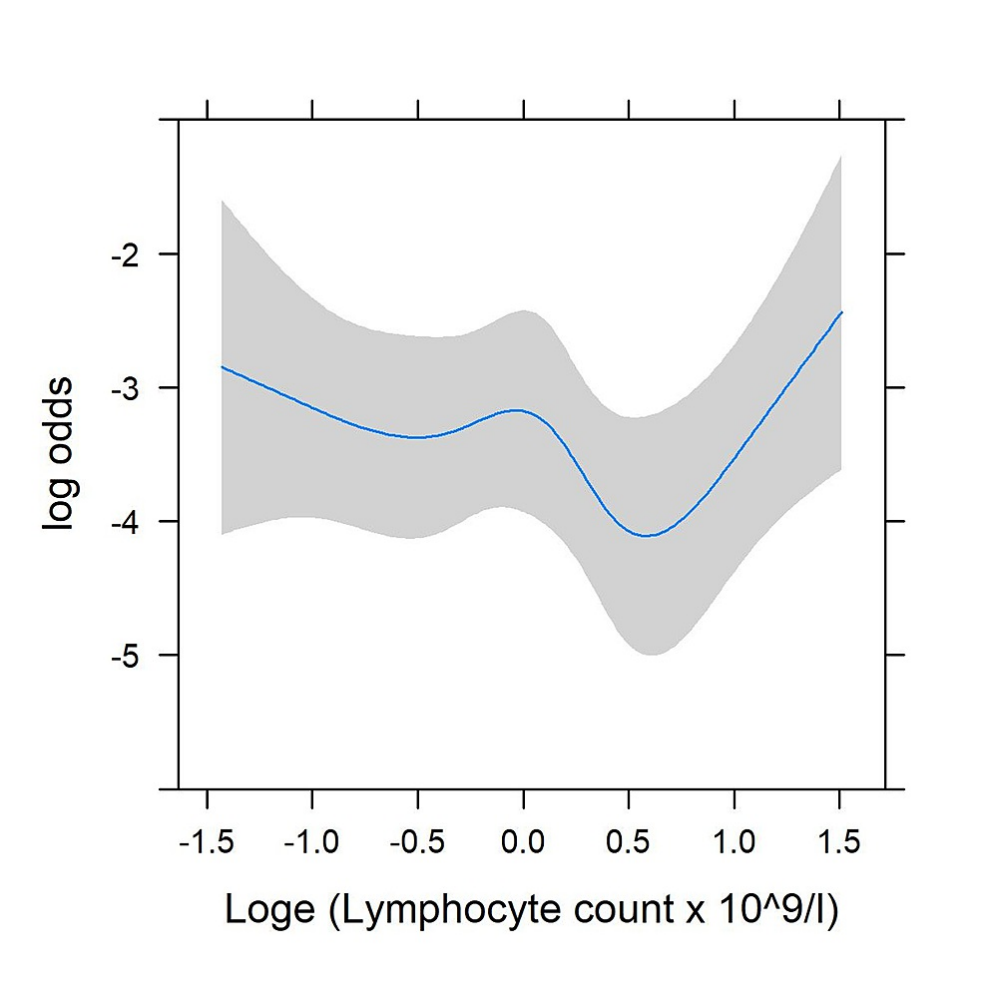
Restricted cubic splines for the non-linear association of lymphocyte count with mortality; adjusted for age, sex, swab positivity, and comorbidity. Log: Logarithm Loge: Exponent of the log

There was significant evidence of non-linearity (p < 0.001) in the unadjusted model, with risk no longer declining once lymphocyte levels increased above 1.6 x 109/L (Figure 1). After adjustment for comorbidity, there was no significant non-linearity (p = 0.13, Figure 2).

**Figure 3:**
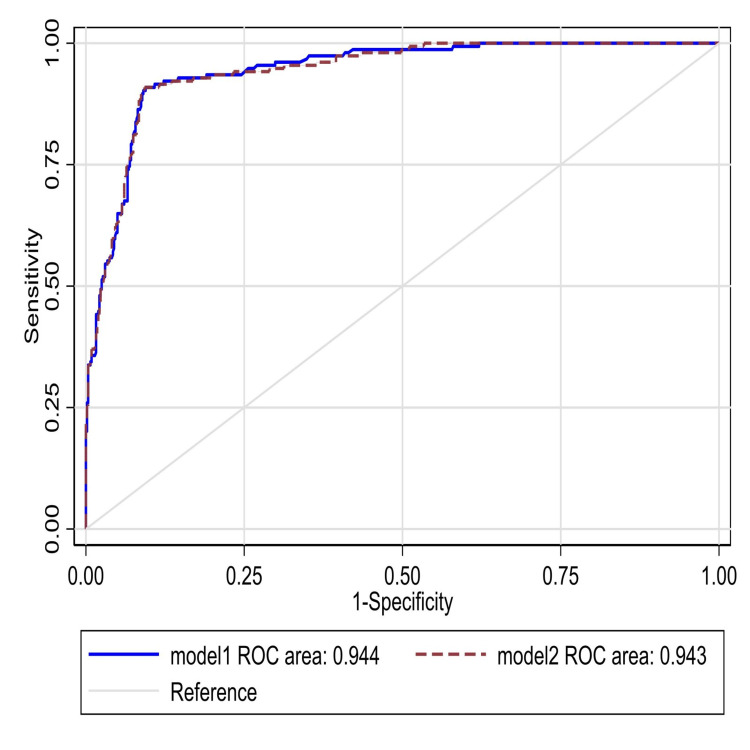
Area under the ROC curve for predictive models for mortality in all patients. Model 1 without lymphocyte count and Model 2 with lymphocyte count added. ROC: Receiver operating characteristic curve.

We also examined whether the effect of lymphocyte count differed by age (Table 5), and found no evidence of interaction (p = 0.09). Lasso model was used to analyze mortality in swab-positive patients (Table 6) and overall mortality (Table 7). Finally, this data was analyzed for the association of lymphocyte count and without lymphocyte count for any relevance with the area under a receiver operating characteristic (ROC) curve (Figure 3).

**Table 5:** Lymphocyte count by mortality and quartile of age: median [IQR] N. IQR: Interquartile range; N: Number of cases; P: Probability of obtaining results.

	All patients		Positive patients	
Age, years	Alive	Deceased	Alive	Deceased
N	N = 584	N = 158	N = 74	N = 38
<58 years	1.63 [1.12-2.04] 179	0.76 [0.56-1.46] 7	1.36 [1.03-1.87] 28	0.56 [0.56-0.56] 1
58-72 years	1.32 [0.84-1.76] 154	0.74 [0.54-1.15] 35	0.99 [0.66-1.40] 20	1.15 [0.73-4.02] 9
73-84 years	1.07 [0.71-1.33] 132	0.72 [0.42-1.05] 58	0.71 [0.47-1.16] 8	0.75 [0.54-0.78] 14
>=85 years	0.87 [0.55-1.28] 119	0.85 [0.59-1.08] 58	1.05 [0.62-1.37] 18	0.93 [0.59-1.28] 14
P-value (interaction)	P = 0.09		P = 0.66	

**Table 6:** Lasso models for predicting death in swab-positive patients. Lymphocyte does not contribute to the predictive model for swab-positive patients. COPD: Chronic obstructive pulmonary disease; Frailty: Dalhousie frailty (Rockwood) score of 4 or more classified as frail; IHD: Ischemic heart disease; ILD: Interstitial lung disease; ROC: The area under a receiver operating characteristic curve.

Variable	Model coefficients
Age	0.012
IHD	2.943
Asthma/COPD/ILD	3.066
Frailty	2.086
ROC area (95% CI)	0.981 (0.962-1.00)

**Table 7:** Lasso models for predicting death – all patients. COPD: Chronic obstructive pulmonary disease; Frailty: Dalhousie Frailty (Rockwood) score of 4 or more classified as frail; IHD: Ischemic heart disease; ILD: Interstitial lung disease; ROC: Area under a receiver operating characteristic curve.

	Model 1	Model 2
Variable	Model coefficients	Model coefficients
Age	0.027	0.022
Male sex	0.528	0.429
Swab positive	0.256	0.230
IHD	2.145	2.057
Asthma/COPD/ILD	1.902	1.796
Hypertension	0.493	0.455
Dementia	-0.105	-
Frailty	2.112	2.050
Malignancy	4.816	4.381
Lymphocyte	-	-0.198
ROC area (95% CI)	0.944 (0.926-0.962)	0.943 (0.924-0.961)

## Discussion

COVID-19 is a highly infectious disease with significant morbidity and mortality. Although there is no relative scarcity of information towards awareness for the severity of the disease, there remains a lot of need to do more to improve the overall outcome. Multiple biomarkers have been considered towards its diagnosis along with COVID-19 swabs, which have been noted as having low sensitivity [9]. The utilization of lymphocyte count and in particular lymphopenia has drawn great attention for practice towards managing patients suspected to have COVID-19 [1,2]. Other studies have focused on the association in patients admitted to the ICU [3] or the odds of getting admitted to the ICU with lymphopenia [10]. There is a trend towards a higher risk of acquiring infection as well as increased severity with COVID-19 in absolute lymphopenia range. The presence of lymphopenia is associated with a threefold increased risk of severe COVID-19 [10].

Others have studied the association of lymphocyte and neutrophil count and COVID-19 [11]. Yun H et al. reported that lymphocyte counts were markedly low in COVID-19-positive patients [12]. Similarly, a recently published study from Wuhan, China, showed that the lymphocyte count was considerably lower among patients who succumbed to COVID-19 compared to those who survived [13]. On the contrary, our study shows no relationship between lymphopenia and having COVID-19.

Furthermore, in COVID-19 patients, mortality rates did not change with the level of lymphocyte count. Any observed association within the total population was explained by the increased prevalence of comorbidities among those with lower lymphocyte count. Interestingly, it has long been determined that the process of lymphopoiesis is noticeably disrupted in the aging population due to an aging immune system. Since our study cohort includes patients of all age groups rather than merely the elderly, it is possible that we did not see any relationship with lymphopenia [14,15]. Although even in older patients we found no association of lymphocyte count and COVID-19 infection or mortality with COVID-19. Based on the statistical analysis of comorbidity data, it appears that comorbidities contribute more towards mortality than lymphocyte count.

Our study does have limitations, including no standardization for ethnicity and inclusion of only acute presentations. Patients who were referred to our hospitals for swab testing but had no lymphocyte count performed were excluded. Patients who had a COVID-19 swab test but were not admitted to the hospital were also excluded as their blood lymphocyte levels were not available.

As our analysis is conditional on patients having a test, we cannot rule out selection bias in our results. However, the observed associations with testing positive and mortality were all in the expected direction. As the world attempts to grasp the COVID-19 phenomenon, we realize a number of observations have been made towards associations [16] and emerging evidence of side-effects of varying COVID-19 vaccines [17]. We hope our study offers the missing piece of the puzzle towards comorbidity associations.

## Conclusions

In conclusion, we did not find a strong relationship between lymphocyte levels and COVID-19-related mortality, especially the mortality caused by the infection, with age, gender, swab outcomes, and comorbidities adjusted. Additionally, there was no association with testing positive, as swab-positive patients did not show a significant reduction in lymphocyte compared to swab-negative patients. The most vulnerable group of the population for COVID-19, the aging population, as well as other age groups, have factors aside from lymphocyte levels, which may include comorbidities such as diabetes mellitus type 1 and 2; various cancers, and chronic heart failure. These comorbidities may contribute to more association with acquiring infection and increased mortality with COVID-19. Overall, this study brings to light the misconception of one of the more widely embraced myths that low lymphocyte count is a major predisposing factor in getting this virus.

## Appendices

Supporting Information:

Appendix 1:

Authors contribution:

Dr. Mansoor Zafar designed the study and wrote the manuscript.

Ms. Jackie Cooper assisted in the statistical analysis of the study.

Steve Moran and William A. O'Neill contributed to the provision of electronic COVID-19 swab outcomes for the two hospitals.

Dr. Mansoor Zafar, Dr. Muhammad Shahbaz, Dr. Mangala Karkhanis, Dr. Mohamed Abdelbagi, Dr. Opeyemi A. Makanjuola, Dr. Bipin Pun, Dr. Ratan S. Randhawa, Dr. Frederic Cuison, Dr. Dana Safarova, Dr. Oluwamayowa Ojofeitimi, Dr. Kamal Lawrence, Dr. Mariya Farooq, Dr. Reem Eldebri, Dr. Saba Alam, Dr. Lucinda Barry, Dr. Alisha Khanna, Dr. Karuna Subba, Dr. Amr Elyasaky, Dr. Hesam A. Nooredinvand, Dr. Manivannan Periasamy, Dr. Bolurin A. Adekunle, Dr. Zahra Maryam, Dr. Bao Khuu, Dr. Johannes Hegner, Dr. Andreia Esteves Morete, Mr. Mirej Patel, Dr. Gjulio Ciroi, Dr. Ubaid Ur Rehman, and Bianca A. Lazau assisted with electronic records of the patients.

Dr. Mansoor Zafar, Dr. Muhammad Shahbaz, Dr. Mangala Karkhanis, Dr. Viktoriya Clarke, Dr. Athanasios Nakos, Dr. Tila Muhammad, and Dr. Stefano Berliti assisted with proofreading the manuscript.

Dr. Saba Alam, Jabeen Hsiao, Ms. Maaryah J. Zafar, Ms. Nadiyah Zafar, and Dr. Najam-us-Saher Rizvi assisted with cross-checking data entry for any errors and help with the references.

Dr. Umesh Dashora, Dr. Simon Merritt, Dr. Ellie M. Giddings, Dr. Mark Whitehead, and Dr. Osei Kankam reviewed the manuscript.

29+ contributors helped in collecting the data. All authors/contributors read and approved the final manuscript.
